# Comparative Analysis of Tumor Characteristics, Treatment Response, and Oncological Outcomes in Early-Onset Versus Late-Onset Colorectal Cancer: A Retrospective Cohort Study

**DOI:** 10.7759/cureus.82975

**Published:** 2025-04-25

**Authors:** Muhammad S Khan, Azwa Ali, Murk Niaz, Raheena Hassan, Bushra Shirazi, Raja Taha Yaseen Khan

**Affiliations:** 1 Department of General Surgery, Sindh Institute of Urology and Transplantation, Karachi, PAK; 2 Department of Hepatogastroenterology, Sindh Institute of Urology and Transplantation, Karachi, PAK

**Keywords:** early onset colorectal cancer, late onset colorectal cancer, screening, survival outcomes, tumor aggressiveness

## Abstract

Introduction: Colorectal cancer is a leading cause of cancer-related mortality worldwide, with distinct clinical features observed between early-onset colorectal cancer (EOCRC, ≤50 years) and late-onset colorectal cancer (LOCRC, >50 years). Recent trends indicate an alarming rise in EOCRC incidence, underscoring the importance of understanding its unique tumor biology and clinical behavior. Therefore, the aim of this study is to compare the tumor characteristics, clinico-pathological features, and oncological outcome between EOCRC and LOCRC.

Study methodology: This retrospective cohort study evaluated 162 patients with histopathologically confirmed colorectal adenocarcinoma who underwent treatment with curative intent at Sindh Institute of Urology and Transplantation, Karachi, Pakistan, between August 2018 and July 2023. Patients were stratified into EOCRC (n=78) and LOCRC (n=84) groups. Clinical data, including demographics, tumor location, histological differentiation, staging, treatment modalities, and recurrence patterns, were extracted from medical records and analyzed using chi-square tests and Kaplan-Meier survival estimates.

Results: EOCRC patients more frequently presented with rectal tumors, poorly differentiated histology, and advanced stage disease at diagnosis. Although pathological staging and resection margins were comparable, the EOCRC group exhibited a significantly higher incidence of distant recurrence and lower rates of complete pathological response to neoadjuvant chemoradiotherapy. At 24 months, both disease-free and overall survival were markedly lower in EOCRC compared to LOCRC patients.

Conclusion: The study demonstrates that EOCRC is characterized by aggressive tumor behavior and poorer oncological outcomes. These findings support the need to revise screening strategies, such as lowering the age threshold and incorporating flexible sigmoidoscopy, to enhance early detection and improve patient prognosis.

## Introduction

Colorectal cancer, the most common gastrointestinal malignancy, is the third most common malignancy and fourth leading cause of cancer-related deaths worldwide [1]. Age-related disparity in colorectal cancer presentation and outcome leads to the division of this cancer into two different subgroups. Early-onset colorectal cancer (EOCRC) refers to the cases diagnosed at the age of ≤50 years, while late-onset colorectal cancer (LOCRC) refers to the cases diagnosed after 50 years of age [2].

Incidence and mortality related to colorectal cancer have been decreased worldwide, especially in patients aged more than 50 years, and this is most likely due to the development of screening programs for this age group in the general population [3]. However, recently there has been a change in the trend of EOCRC, with a rise in incidence from 2-4% in 2012 to 7-14% in 2016, requiring an aggressive course of chemotherapy, and is associated with a mixed overall prognosis [4]. A systematic review of 40 studies across 12 countries and five continents reported a 30% increase in the incidence of EOCRC over the past 20 years [5]. Literature review indicates that EOCRC has a large proportion of sporadic onset, while hereditary cancer in this age group accounts for 30% of the total [6].

Several studies have been conducted to evaluate the possible risk factors other than genetic causes responsible for the development of EOCRC. A meta-analysis examining 20 studies through MEDLINE and database search was conducted in this regards and concluded that apart from general risk factors like gender, smoking, alcoholism, family history and body mass index (BMI) some other potential risk factors specially related with EOCRC include central obesity, hyperlipidemia, sedentary life style, occupation, junk food and hypertension [7].

The disease presentation, clinico-pathological characteristics, response to treatment, and outcome of patients with EOCRC are significantly different when compared with LOCRC patients. Current literature identified that EOCRCs are more common in the left colon and rectum, while LOCRCs are more marked in the right-sided colon [8]. Comparative studies between EOCRC and LOCRC demonstrate that EOCRCs are more aggressive and locally advanced at the time of diagnosis, with a high rate of signet ring cell component, poorly differentiated, and mucinous histology. Hence, the survival outcome and response to neo-adjuvant treatment are also compromised [9].

A better understanding of tumor characteristics, clinico-pathological features, and disease outcome is the key factor that can help to identify the difference between EOCRC and LOCRC [10]. This will help to establish an appropriate treatment strategy for a different set of colorectal cancer patients, which will ultimately be beneficial to overcome differences in tumor behavior and response to treatment and oncological outcome [11,12].

There is a lack of local data that compares the demographics and clinical characteristics between the EOCRC and LOCRC. Therefore, the aim of this study is to compare the tumor characteristics, clinico-pathological features, and oncological outcome of two different sets of colorectal cancer patients (EOCRC vs. LOCRC) and to evaluate age of disease onset as an independent risk factor to predict oncological outcome, including disease progression, recurrence, and survival.

## Materials and methods

Study design

A retrospective study was conducted in the Department of General Surgery, Sindh Institute of Urology and Transplantation, Karachi, Pakistan, on patients diagnosed with colorectal adenocarcinoma. All the patients diagnosed with histopathologically proven colorectal adenocarcinoma treated with curative intent from August 2018 till July 2023 were enrolled in the study. However, patients having locally advanced, unresectable, or metastatic disease, patients who were lost to follow-up, or patients in whom treatment was not completed were excluded from the study.

Data collection procedure

The records of all the patients were retrieved from the Hanifa Suleiman Oncology clinic registry, GI clinic patients' registry, histopathology department, and hospital record room. Variables mentioned in the questionnaire were all collected and stored with a given medical record number, maintaining data security and confidentiality. Approval for the study and exemption letter was obtained from the Institutional Review Board (IRB) and Ethical Review Committee (ERC) (approval no.: SIUT-ERC-2024/A-929). All variables mentioned in the questionnaire were collected from the patient’s files including age, gender, smoking history, endoscopy (colonoscopy/sigmoidoscopy) findings, histological tumor differentiation, site of tumor, clinical and pathological tumor, node, and metastasis (TNM) classification, neoadjuvant/adjuvant treatment given or not, surgical procedure performed, follow-up, tumor recurrence, and overall survival.

All the patients were divided into two groups. Group A comprised those patients with EOCRC (age ≤50 years), while group B comprised those with LOCRC (age ≥51 years). The treatment protocol was standardized for all patients based on disease stage, with no difference based on patient age. Patients with colonic cancers were operated on upfront, and adjuvant chemotherapy was offered to patients based on tumor pathological stage. However, in cases of rectal cancer, neo-adjuvant chemoradiotherapy was given to all the patients as a standard protocol, followed by tumor resection and adjuvant chemotherapy based on tumor stage.

Data analysis procedure

All data collected were analyzed using IBM SPSS Statistics for Windows, Version 23 (Released 2016; IBM Corp., Armonk, New York, United States). Frequencies and percentages were computed for categorical variables, while mean ± standard deviation (SD) was used for continuous variables. Chi-square was used for the comparison of categorical variables. To estimate survival and recurrence, the Kaplan-Meier method was applied, while the Log-rank test was used for the analysis of survival differences. Statistical significance was defined as a two-tailed p-value of less than 0.05.

## Results

A total of 162 patients with biopsy-proven colorectal adenocarcinoma underwent definite tumor resection surgery in a five-year period from August 2018 to July 2023. Seventy-eight patients (48.1%) fell in the EOCRC group while 84 patients (51.8%) were in the LOCRC group. Thirty-nine patients (50%) of the EOCRC and 49 patients (58.3%) of the LOCRC group were male. Most of the patients from the EOCRC group, that is 89.7%, were non-smokers. Colonic cancer was reported in 22 patients (28.2%) of EOCRC and 46 patients (54.7%) of LOCRC group, while rectal cancer was observed in 56 patients (71.7%) of EOCRC and 38 patients (45.2%) of LOCRC group. These results indicate that the EOCRC group had a larger proportion of rectal cancer patients when compared with the LOCRC group. Subgroup analysis of colonic cancer revealed that a large proportion of the LOCRC group (39 patients, 46.4%) had right colonic cancer, while 12 patients (15.3%) from the EOCRC group had right colonic cancer. A major proportion of EOCRC patients (44.8%) had poorly differentiated tumors compared to 11.9% of patients from the LOCRC group, showing worse histological features in the EOCRC group. In our study, none of the patients from the EOCRC group had stage I cancer on clinical presentation (cTNM), while 17.8% of patients from the LOCRC group had stage I disease. On the contrary, 71.7% of patients from the EOCRC group had stage III disease compared to 53.5% of the LOCRC group, showing that the EOCRC group has advanced disease at initial presentation (Table 1).

**Table 1:** Clinical parameters of the studied population (n=162) * The chi-square test was used for the calculation of p-values. Values are presented as frequency (percentage). EOCRC: Early-onset colorectal cancer; LOCRC: Late-onset colorectal cancer; TNM: Tumor classification according to the tumor, involvement of lymph nodes, and presence of local or distant metastasis; APR: Abdominoperineal resection; LAR: Lower anterior resection; AR: Anterior resection

Parameters		EOCRC (n=78, 48.1%)	LOCRC (n=84, 51.8%)	Chi-square value*	P-value
Gender	Male	39 (50%)	49 (58.3%)	1.13	0.726
	Female	39 (50%)	35 (41.6%)		
Smoker	No	70 (89.7%)	65 (77.3%)	4.45	0.004
	Yes	8 (10.2%)	19 (22.6%)		
Tumor location	Right colon	12 (15.3%)	39 (46.4%)	27.9	0.003
	Left colon	10 (12.8%)	2 (2.3%)		
	Sigmoid colon	0 (0)	5 (5.9%)		
	Rectum	56 (71.7%)	38 (45.2%)		
Tumor differentiation	Well	21 (26.9%)	38 (45.2%)	21.9	0.013
	Moderate	22 (28.2%)	36 (42.8%)		
	Poor	35 (44.8%)	10 (11.9%)		
Tumor biology	Mucinous	17 (21.7%)	7 (8.3%)	18.8	0.011
	Signet ring	22 (28.2%)	8 (9.5%)		
	Adenocarcinoma	39 (50%)	69 (82.1%)		
Clinical stage (cTNM)	I	0 (0)	15 (17.8%)	16.1	0.031
	II	22 (28.2%)	24 (28.5%)		
	III	56 (71.7%)	45 (53.5%)		
Surgical procedure	APR	32 (41%)	14 (16.6%)	31.6	0.009
	LAR/AR	20 (25.6%)	21 (25%)		
	Sigmoidcolectomy	0 (0)	5 (5.9%)		
	Hartman procedure	4 (5.1%)	3 (3.5%)		
	Left hemicolectomy	10 (12.8%)	2 (2.3%)		
	Right hemicolectomy	12 (15.3%)	39 (46.4%)		

Results from pathological TNM staging were comparable between the two groups, as well as circumferential resection margins (CRM) status, perineural invasion, and lymphovascular invasion were all comparable with no statistically significant difference reported between the two groups. Eighteen patients (23%) from the EOCRC group, while 10 patients (11.9%) from the LOCRC group had local recurrence, although this is not statistically significant, but the number is almost double for EOCRC patients. Distant recurrence was reported in 16 patients (20.5%) from EOCRC and in four patients (4.7%) from the LOCRC group, which is statistically significant. Proximal and distal resection margins of the tumor were clear in all patients. Pathological complete response was observed in patients with rectal cancer who underwent neo-adjuvant chemoradiotherapy before definite surgical resection of the tumor, and it was observed that only nine patients (11.5%) from the EOCRC group had complete pathological response compared to 24 patients (28.5%) from the LOCRC group. These results showed the aggressive nature of the disease in the EOCRC group with limited response to neo-adjuvant treatment, however, these results were statistically not significant (Table 2).

**Table 2:** Comparison of post-treatment responses in early and late onset CRC groups *The chi-square test was used for the calculation of p-values. Values are presented as frequency (percentage). EOCRC: Early-onset colorectal cancer; LOCRC: Late-onset colorectal cancer; TNM: Tumor classification according to the tumor, involvement of lymph nodes, and presence of local or distant metastasis; CRM: Circumferential resection margins; PNI: Perineural invasion; LVI: Lymphovascular invasion

Parameters		EOCRC (n=78, 48.1%)	LOCRC (n=84, 51.8%)	Chi-square value*	P-value
Pathological stage (pTNM)	0	4 (5.1%)	7 (8.3%)	7.5	0.157
	I	6 (7.6%)	17 (20.2%)		
	II	29 (37.1%)	20 (23.8%)		
	III	39 (50%)	40 (47.6%)		
CRM	Negative	69 (88.4%)	79 (94%)	1.6	0.294
	Positive	9 (11.5%)	5 (5.9%)		
PNI	Negative	63 (80.7%)	58 (69%)	2.9	0.182
	Positive	15 (19.2%)	26 (30.9%)		
LVI	Negative	44 (56.4%)	48 (57.1%)	0.009	0.568
	Positive	34 (43.5%)	36 (42.8%)		
Local recurrence	No	60 (76.9%)	74 (88%)	3.53	0.271
	Yes	18 (23%)	10 (11.9%)		
Distant recurrence	No	62 (79.4%)	80 (95.2%)	9.3	0.042
	Yes	16 (20.5%)	4 (4.7%)		
Complete response to neoadjuvant therapy for rectal cancer only	Yes	9 (11.5%)	24 (28.5%)	7.2	0.093
	No	69 (88.4%)	60 (71.4%)		

**Figure 1:**
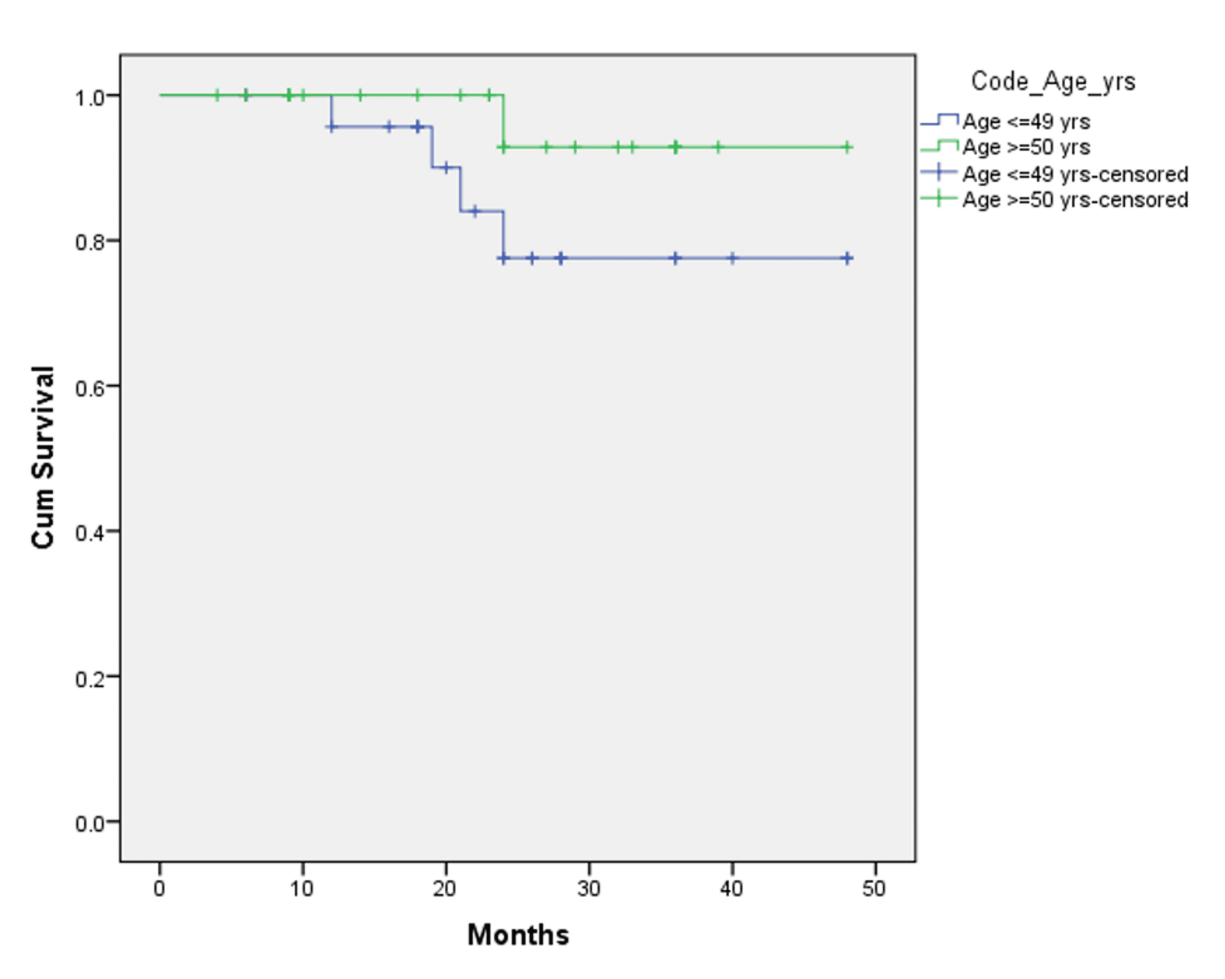
Comparison of overall survival in EOCRC and LOCRC EOCRC: Early-onset colorectal cancer; LOCRC: Late-onset colorectal cancer

**Figure 2:**
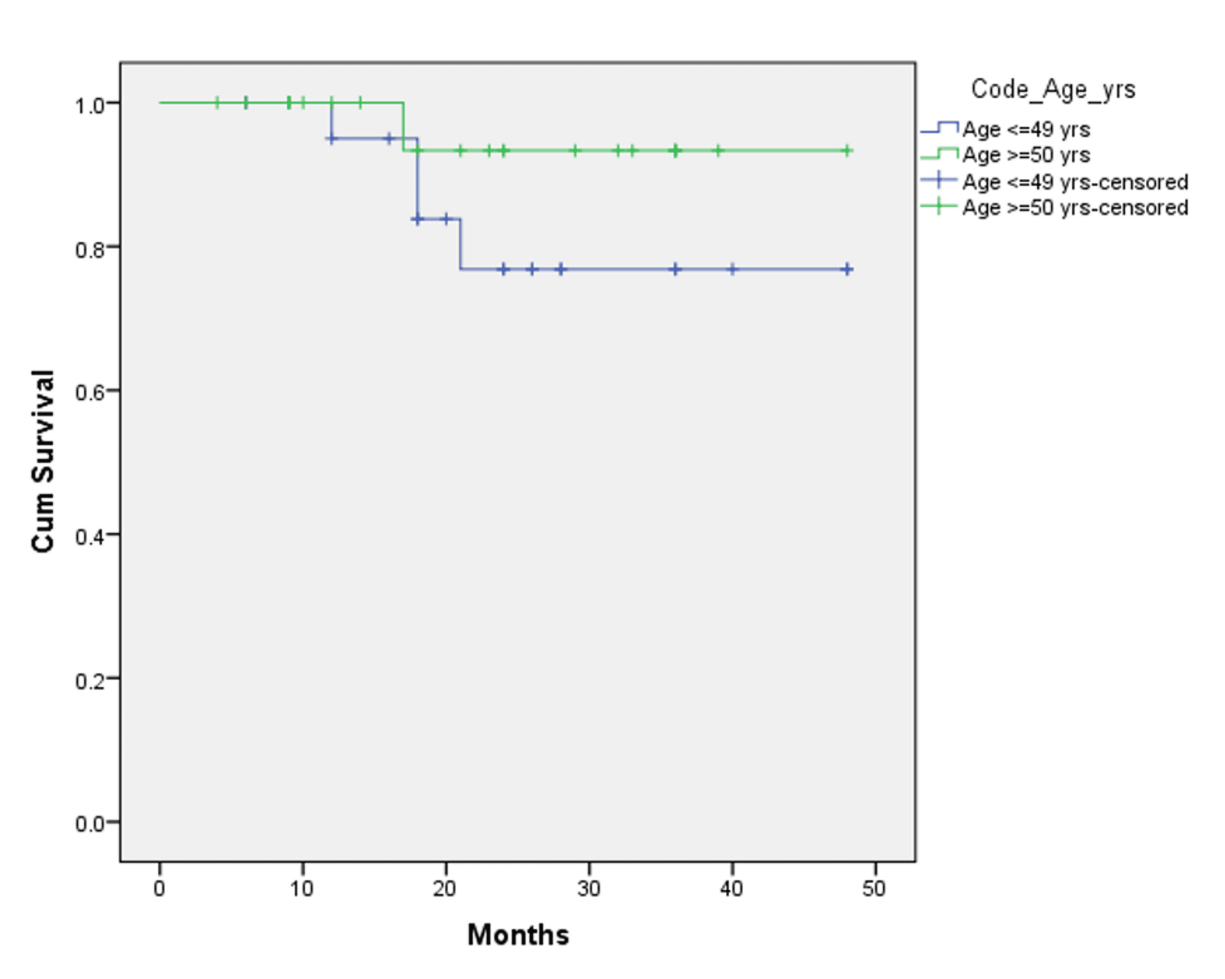
Disease-free survival and recurrence in EOCRC and LOCRC EOCRC: Early-onset colorectal cancer; LOCRC: Late-onset colorectal cancer

Two years after surgery, the results of the two groups were analyzed using the Kaplan-Meier curve, and the results showed that at 24 months follow-up, 76.8% of patients were disease-free in the EOCRC group as compared to 93.3% of patients in the LOCRC group. The recurrence (both local and distant combined) reported in the EOCRC group was 23.2%, while that in the LOCRC group was 6.7% (Figure 1). At 24 months follow-up, 77.6% of patients were alive (with or without disease) in the EOCRC group compared to 92.9% of patients in the LOCRC group (Figure 2).

## Discussion

Age-related disparity in colorectal cancer patients has been evident from various studies worldwide, and multiple differences between the two groups (EOCRC vs. LOCRC) have been highlighted, including clinicopathological features, tumor location, response to neo-adjuvant therapy, and oncological outcome [13].

Early onset cancers are more marked with their advanced nature at initial presentation, and this is attributed to the delay in diagnosis, which is considered a major factor for the advanced nature of the disease. Various factors have been studied for the delay in diagnosis of cancer in the early onset group, which include low suspicion of cancer in this age group by general clinicians, lack of knowledge and understanding of symptoms of this disease, which overlap with usual stomach upset symptoms. Patient-related factors also contribute to delay presentation and hence advanced disease, which include fear, denial, and absence of disease information, financial issues, and difficulty accessing healthcare providers in remote areas, especially in low- and middle-income countries [14].

Internationally reported data specify that patients with EOCRC presented with advanced disease stage III and stage IV at initial presentation, which is in accordance with the data reported in our study, including the major proportion of the patients present with stage III disease while none of the patient had stage I disease at initial presentation [15]. Lack of screening program in this age group is one of the contributing factors for delay in diagnosis, however, aggressive histopathological characteristics of the disease in the early age group are the major contributing factor.

Our study also pointed out site-related disparity in the two sets of patients, reporting that EOCRC patients presented with more left-sided colonic and rectal cancer, while LOCRC patients had more right-sided colonic cancers, and these results are similar to what has been published in international literature [12]. Considering this site-related disparity in EOCRC patients and lack of screening program in this age group, introducing flexible sigmoidoscopy as the initial screening tool of the EOCRC age group individuals would definitely reduce screening burden and at the same time increase the probability to capture the majority of EOCRC patients [16].

Aggressive histopathological characteristics, including lympho-vascular invasion, extramural venous invasion, perineal invasion, and node positivity, are the factors responsible for the advanced nature of the disease in EOCRC patients as stated in internationally published literature [17]. However, our study didn’t show any significant difference in the two sets of patients when compared for these parameters. This can be attributed to the demographic characteristics of our population as compared to the rest of the world. On the other hand, poorly differentiated tumors, along with mucinous and signet ring differentiation, which is a well-established factor for compromised survival and early recurrence, and even advanced unresectable disease at initial presentation [9,18], are more marked in the EOCRC group, and the same results were concluded in our study.

Internationally reported data showed controversial results regarding tumor recurrence, disease-free survival, and overall survival in two groups of CRC patients. Some studies show worse outcomes in EOCRC compared to LOCRC [9,19], while other studies show equivalent or even better outcomes [9]. However, age stratification indicates that EOCRC is worse in terms of outcome compared to LOCRC patients [20]. Our results are in accordance with the published data showing worse local and distant recurrence in EOCRC when compared to LOCRC patients, as well as disease-free survival and overall survival are also compromised in the EOCRC group.

In our study, subgroup analysis of rectal cancer regarding pathological response, complete response after neo-adjuvant chemoradiotherapy was observed, and the LOCRC patients showed more complete response on final histopathology as compared to EOCRC patients, specifying that tumor biology and aggressive nature of disease are more prevalent in EOCRC group patients, which leads to compromised response to neo-adjuvant chemoradiotherapy [21]. These results are also highlighted in the available literature, which raises the question of changing the treatment strategy for EOCRC patients shifting from neo-adjuvant chemoradiotherapy followed by surgery to upfront surgery and hence opens a new focus of discussion in this regard [4,21].

Our study has certain limitations. First, its retrospective nature, along with data heterogeneity and variations in treatment options based on tumor stage at initial presentation, may influence the interpretation and generalizability of the findings. This is a single-center study, and due to the lack of a tumor registry in our population, the study may not represent the true population. This study only addresses trends of the disease and does not specify any disease-related association. Second, our study lacked the information on genetic mutations and molecular markers, which would have helped us in identifying the exact genes responsible for EOCRC and in improving.

The strength of the study is that this study strictly defines the age limit of the two sets of patients (EOCRC vs. LOCRC). This study will develop colorectal cancer awareness in general practitioners and promote the screening-related information for underdeveloped, resource-strained countries like Pakistan. It also considers the flexible sigmoidoscopy as a tool for screening, specifically for a population aged less than 50 years.

## Conclusions

Young patients diagnosed with EOCRC are more likely to present with aggressive tumor biology and advanced-stage disease at the time of diagnosis, compared to their late-onset counterparts (LOCRC). These patients exhibit poorer histological features, higher rates of local and distant recurrence, and reduced disease-free and overall survival. Furthermore, their response to neo-adjuvant therapy, particularly in rectal cancer, is significantly compromised. These findings underscore the need to reevaluate current screening guidelines by lowering the age threshold for colorectal cancer screening. Implementing flexible sigmoidoscopy as a screening modality for individuals under 50 years of age, particularly in low-resource settings, may help facilitate earlier detection and improved clinical outcomes in this vulnerable age group. However, further studies with large sample sizes are required to validate these results and to delineate the difference in clinical impact and prognosis between EOCRC and LOCRC.
